# Enduracididine, a rare amino acid component of peptide antibiotics: Natural products and synthesis

**DOI:** 10.3762/bjoc.12.226

**Published:** 2016-11-07

**Authors:** Darcy J Atkinson, Briar J Naysmith, Daniel P Furkert, Margaret A Brimble

**Affiliations:** 1 School of Chemical Sciences, The University of Auckland, 23 Symonds Street, Auckland, New Zealand; 2 Maurice Wilkins Centre for Molecular Biodiscovery, The University of Auckland, 3 Symonds Street, Auckland, New Zealand

**Keywords:** amino acid, bacterial resistance, enduracididine, natural products, peptide antibiotics

## Abstract

Rising resistance to current clinical antibacterial agents is an imminent threat to global public health and highlights the demand for new lead compounds for drug discovery. One such potential lead compound, the peptide antibiotic teixobactin, was recently isolated from an uncultured bacterial source, and demonstrates remarkably high potency against a wide range of resistant pathogens without apparent development of resistance. A rare amino acid residue component of teixobactin, enduracididine, is only known to occur in a small number of natural products that also possess promising antibiotic activity. This review highlights the presence of enduracididine in natural products, its biosynthesis together with a review of analogues of enduracididine. Reported synthetic approaches to the cyclic guanidine structure of enduracididine are discussed, illustrating the challenges encountered to date in the development of efficient synthetic routes to facilitate drug discovery efforts inspired by the discovery of teixobactin.

## Review

### Introduction

#### The enduracididines

**Figure 1:**
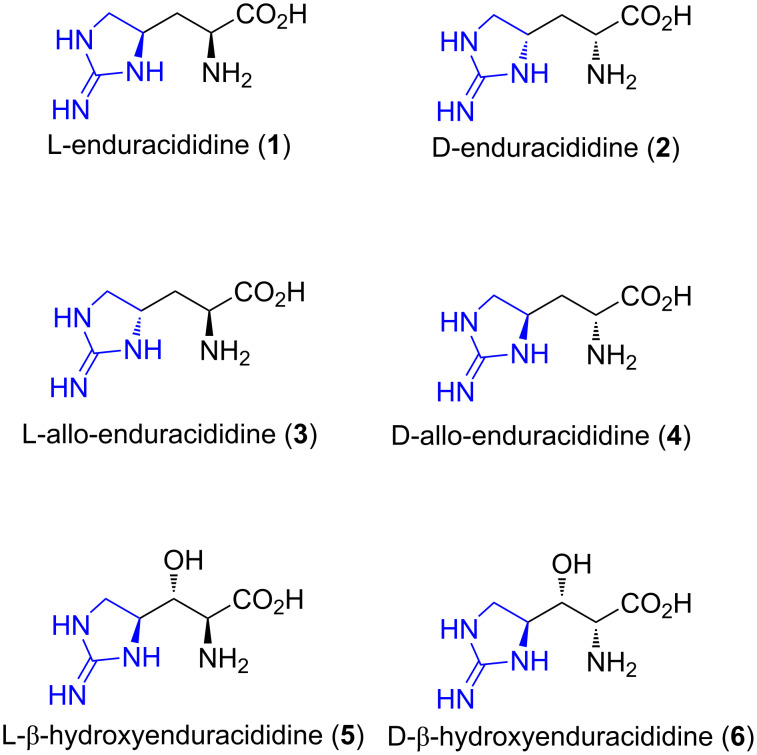
Structures of the enduracididine family of amino acids (**1**–**6**).

The enduracididines (**1**–**6**) are a rare structural class of amino acids that contain a unique five-membered cyclic guanidine moiety (blue, Figure 1). L-Enduracididine (**1**) and D-allo-enduracididine (**4**) were the first identified as amino acid components of potent depsipeptide antibiotics [1–2].

Free enduracididine (**1**) was subsequently isolated from the seeds of the legume *Lonchocarpus sericeus* [3–4]. It was found to inhibit seedling germination of lettuce [5] and did not exhibit any significant effect on the inhibition of protein production in rat hepatoma cells [6]. L-(**5**) and D-β-hydroxyenduracididine (**6**) were first resolved as components of the mannopeptimycin antibiotics, isolated from *Streptomyces hygroscopicus* LL-AC98 in 2002 [7] and to date, have not been isolated as the free amino acids or observed in any other natural products.

### Natural products containing enduracididine and hydroxyenduracididine

#### Enduracidin A and B

**Figure 2:**
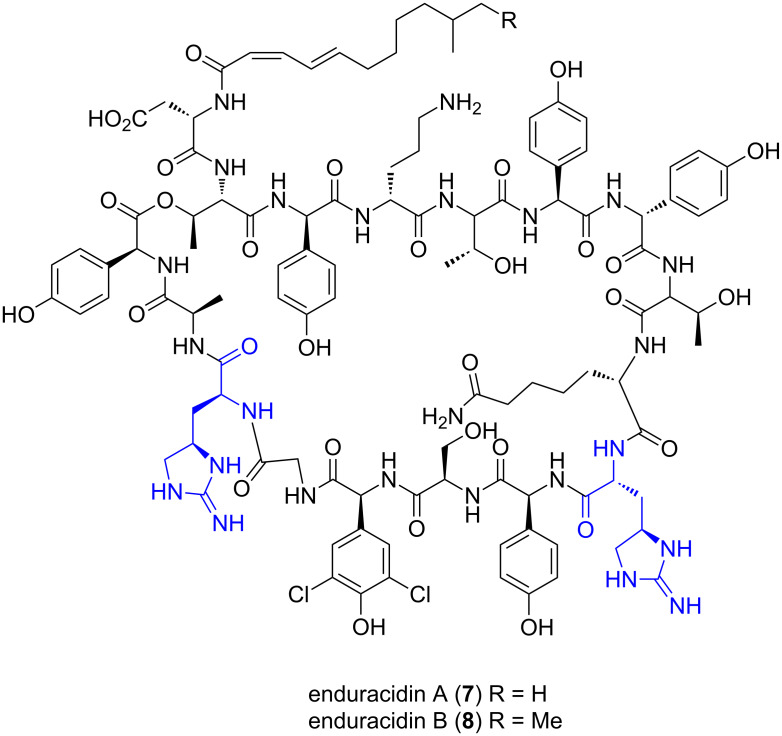
Enduracidin A (**7**) and B (**8**).

Enduracidin A (**7**) and B (**8**) were first isolated from *Streptomyces fungicidicus* B 5477 from a soil sample collected in Nishinomiya, Japan (Figure 2) [1]. Detailed reports of the isolation procedures, in vivo and in vitro antimicrobial activity, physical properties and structure elucidation have been published [1,8–15]. Enduracidin A (**7**) and B (**8**) have also been isolated from *Streptomyces sp*. NJWGY366516 [16], *Streptomyces atrovirens* MGR140 [17] and along with five analogues with various halogenation patterns, from a genetically altered strain of *Streptomyces fungicidicus* [18].

Enduracidin A (**7**) and B (**8**) are depsipeptides with the same composition of seventeen amino acids, sixteen of which make up the cyclic core [11,13,19] and are structurally related to the non-enduracididine containing antibiotic, ramoplanin [19]. The enduracidins are active against Gram-positive bacteria, including resistant strains [2,9,20] and *Mycobacterium* species [21]. No activity was observed against Gram-negative bacteria (except for *Neisseria gonorrhoeae*), fungi or yeast [9]. The antibacterial activity arises through inhibition of cell wall synthesis [22] by prevention of transglycosylation during peptidoglycan synthesis [23], the same step inhibited by vancomycin [24]. Enduracidin A (**7**) and B (**8**) also exhibited inhibition of avian myeloblastosis virus reverse transcriptase but did not suppress replication of HIV cells [25]. Enduracidin A (**7**) and B (**8**) have been produced by fermentation industrially and is used as an antibiotic feed additive for pigs [26] and chickens [27] under the trade name enradin^®^.

#### Minosaminomycin

**Figure 3:**
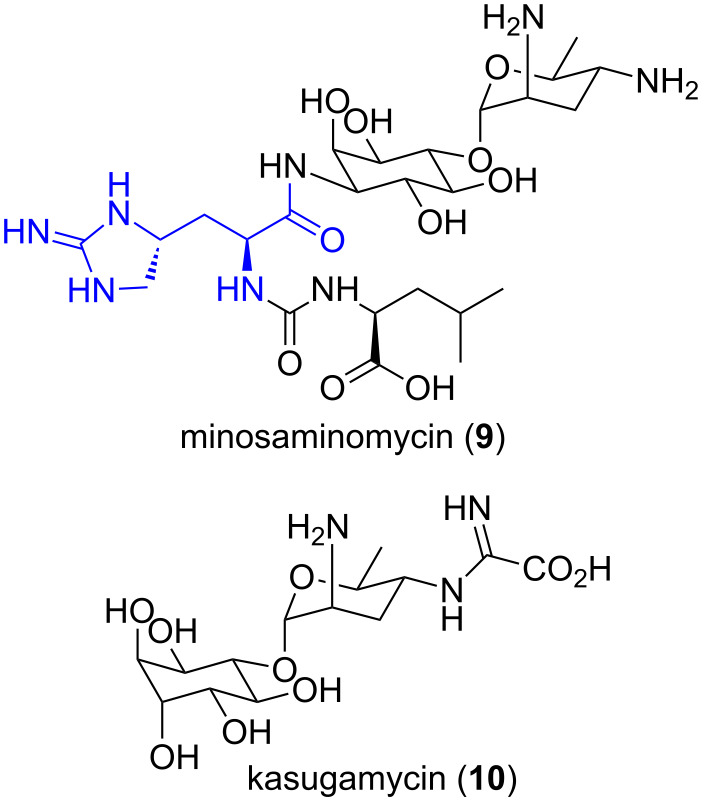
Minosaminomycin (**9**) and related antibiotic kasugamycin (**10**).

Minosaminomycin (**9**, Figure 3) was isolated in 1974 from a culture broth of *Streptomyces* MA514-A1 [28]. It was found to be active against *Mycobacteria* (*M. smegmatis*, MIC = 15.6 μg/mL) but only weakly active against all other bacteria tested. The structure was confirmed through degradation and partial synthesis [29–30]. Minosaminomycin (**9**) inhibits protein synthesis in *E. coli* more effectively than the related antibiotic kasugamycin (**10**), however, a different mechanism is operative [31].

#### Indole metabolite

**Figure 4:**
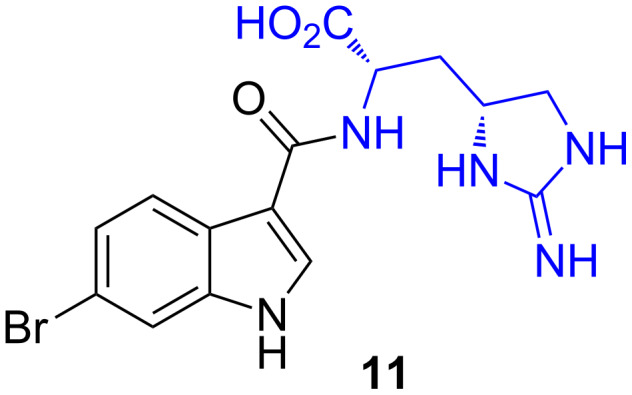
Enduracididine-containing compound **11** identified in a cytotoxic extract of *Leptoclinides dubius* [32].

In 1996, during a screening program for biologically active metabolites from marine ascidians, Riguera et al. identified a small group of amino acid containing compounds in a cytotoxic extract of the ascidian *Leptoclinides dubius* [32]. Among these compounds was the unique enduracididine-containing bromoindole metabolite **11** (Figure 4). This was the first time the enduracididine motif had been isolated from a marine source. The exact compound responsible for the observed cytotoxicity was not determined.

#### Mannopeptimycins

**Figure 5:**
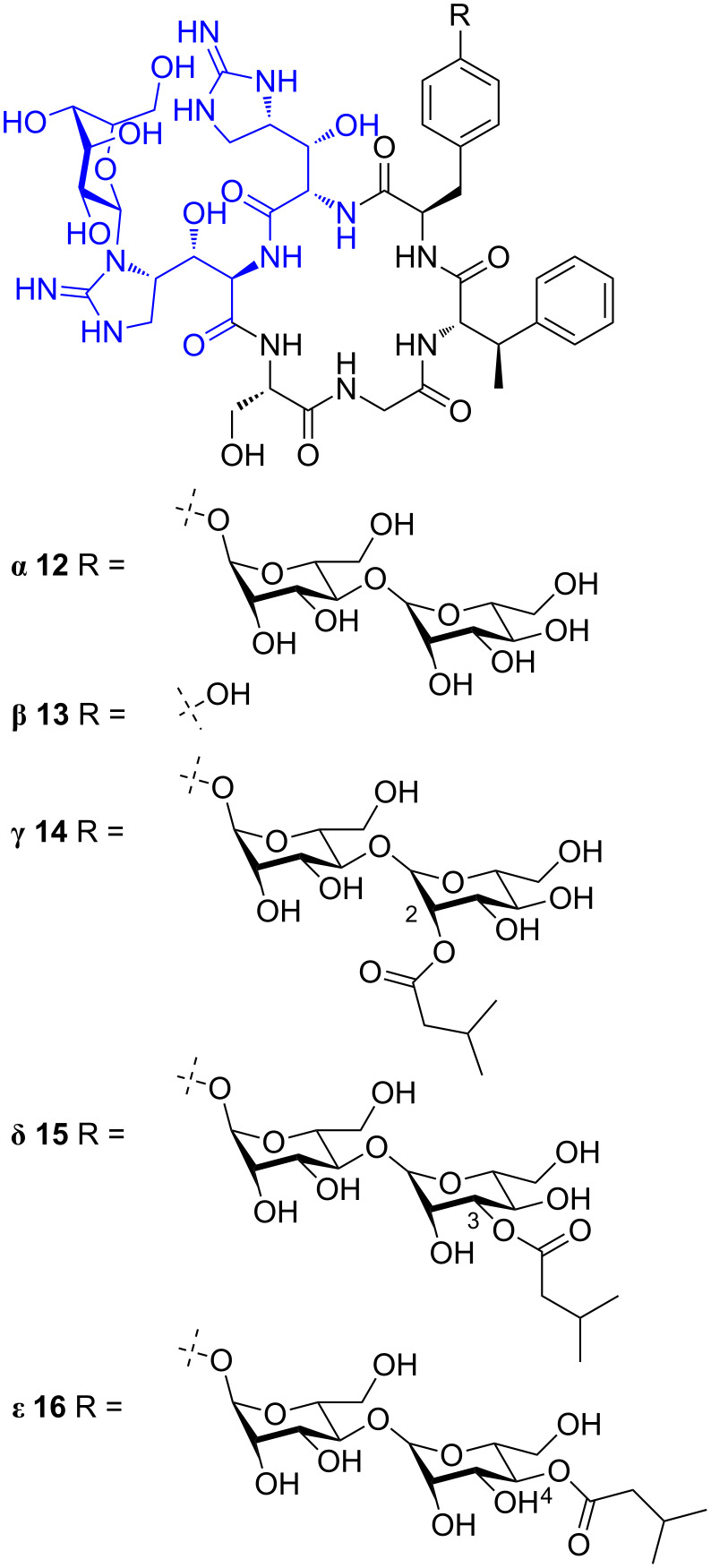
Mannopeptimycins α–ε (**12**–**16**).

Mannopeptimycins α–ε (**12**–**16**, Figure 5) were isolated from *Streptomyces hygroscopicus* LL-AC98 in 2002 [7] and their structures were elucidated using mass spectrometry and extensive NMR analysis. The absolute stereochemistry was proposed following degradation and nOe studies. The configuration of the β-methylphenylalanine stereocentre was revised from *S* to *R*, upon total synthesis [33]. The mannopeptimycins contain a unique sugar substituted hydroxyenduracididine residue (blue, Figure 5).

**Figure 6:**
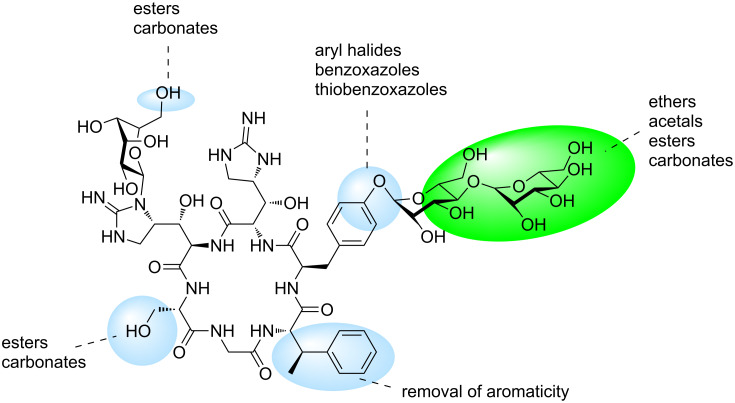
Regions of the mannopeptimycin structure investigated in structure–activity relationship investigations.

The mannopeptimycins displayed moderate activity against Gram-positive bacteria, including MRSA, but only exhibited weak activity against Gram-negative bacteria [7] with the primary cellular target deduced to be bacterial cell wall synthesis [34]. Extensive derivatisation of both mannopeptimycin α (**12**) and β (**13**) was undertaken to improve the antibacterial activity of the parent natural products (highlighted in blue, Figure 6). An array of ether [35–36], halogenated [36], acetal [37–39], benzoxazole [40], thiobenzoxazole [40], ester and carbonate [41] analogues were synthesised and evaluated for antibacterial activity. Only the semisynthetic derivatives possessing hydrophobic groups on the terminal sugar moiety (green) exhibited comparable antibacterial activity to the parent compound and reference antibiotics [42].

#### Teixobactin

**Figure 7:**
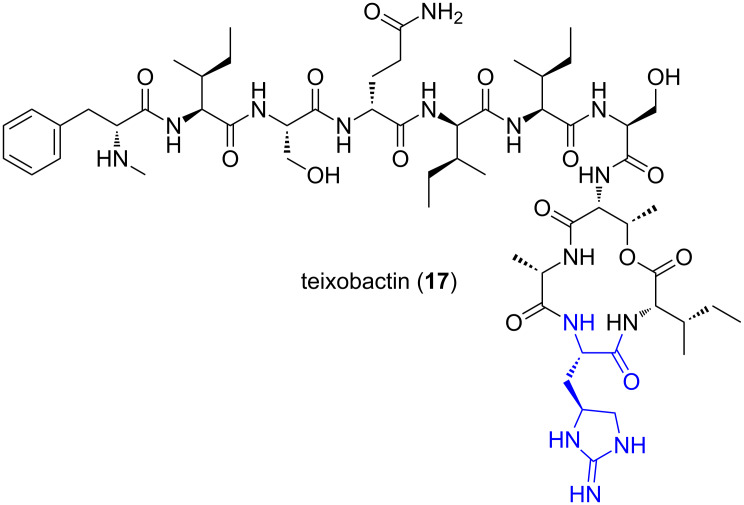
Teixobactin (**17**).

In early 2015, a new enduracididine-containing antibiotic named teixobactin (**17**) was reported (Figure 7) [43]. Teixobactin (**17**) was isolated using the multichannel device, the isolation chip (iChip). The iChip allows a single cell to be delivered to an individual chamber where it can grow. The chambers are covered with a semi-permeable membrane and placed into the microbe’s natural environment where nutrients can diffuse into each chamber. This method gives access to cultures of microbes which were previously unobtainable using traditional techniques. Teixobactin (**17**) exhibits bactericidal activity through binding of Lipid II, a precursor of peptidoglycan, and therefore shows great potential as the foundation for discovery of a new generation of antibiotics to overcome the development of antimicrobial resistance.

Teixobactin (**17**) was isolated from the β-protobacterium, *Eleftheria terrae* that belongs to a new genus. Teixobactin (**17**) demonstrated potent activity against the resistant Gram-positive bacteria, MRSA and vancomycin-resistant *Enterococci* (VRE), as well as other bacterial species including, *Mycobacterium tuberculosis* (Mtb) and *Clostridium difficile*. Remarkably, no teixobactin-resistant mutants of *Staphylococcus aureus* or *M. tuberculosis* could be detected after sub-lethal dosing of the compound over a 27 day period [43]. This lack of resistance development may possibly be attributable to the mechanism of action which involves binding to Lipid II, inhibiting one of the membrane-associated steps of peptidoglycan biosynthesis [43–44]. Analogues of teixobactin (**17**) have undergone biological testing and results show that the L-allo-enduracididine (**3**, blue, Figure 7) residue is important for potent antibacterial activity [45]. An approximately 10-fold reduction in activity was observed when the enduracididine residue is substituted for L-arginine [46] and almost complete loss of activity was observed when three of the four D-amino acids of this analogue are substituted for their L-counterparts [47].

### Biosynthesis of enduracididine

**Scheme 1:**
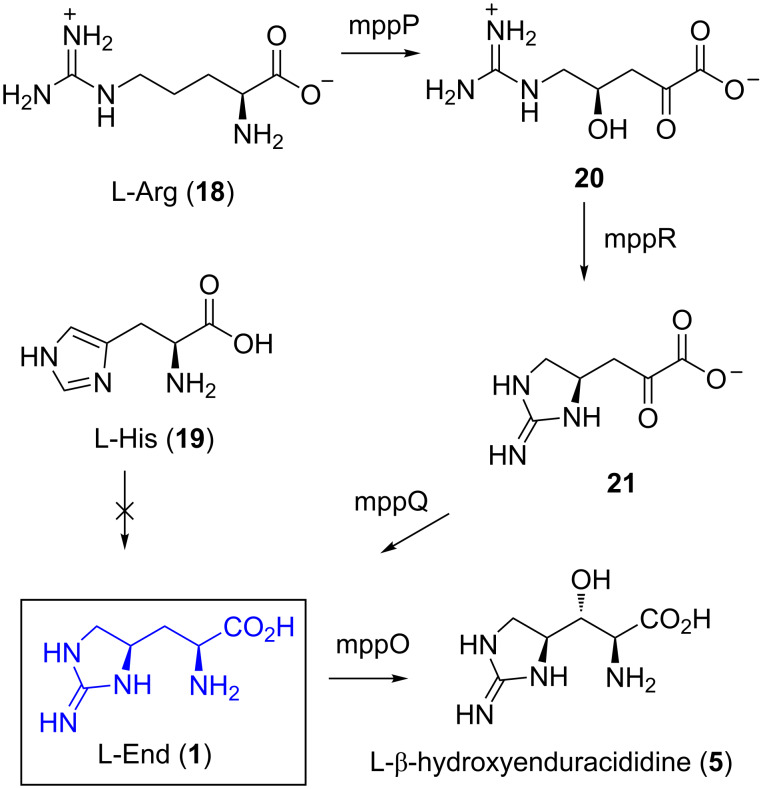
Proposed biosynthesis of L-enduracididine (**1**) and L-β-hydroxyenduracididine (**5**).

In 1984, a radio-labelling study was carried out to determine the biosynthesis of enduracididine (**1**) [48]. Arginine (**18**) and its precursors ornithine and citrulline, were found to be incorporated into enduracididine (**1**), but not histidine (**19**) [48]. Between the enduracidin and mannopeptimycin gene clusters, three pairs of enzymes were found to have high sequence homology, mmpP/endP, mppR/endR and mmpQ/endQ [49–50]. MppP is a PLP-dependent hydroxylase and catalyses the conversion of L-arginine (**18**) and molecular oxygen to 2-oxo-4-hydroxy-5-guanidinovaleric acid (**20**, Scheme 1) [51]. The enzyme mppR is a pyruvate aldose that catalyses the dehydration/cyclisation of **20** to give cyclic guanidine **21** [52], where transamination by mppQ gives enduracididine (**1**). Further transformation to L-β-hydroxyenduracididine (**5**) is then catalysed by mppO [52–53].

### Synthetic investigations

#### Synthesis of enduracididine

Although several synthetic approaches to enduracididine and its derivatives have been published, the discovery of teixobactin (**17**) has reignited interest in the synthesis of this unnatural amino acid.

**Scheme 2:**
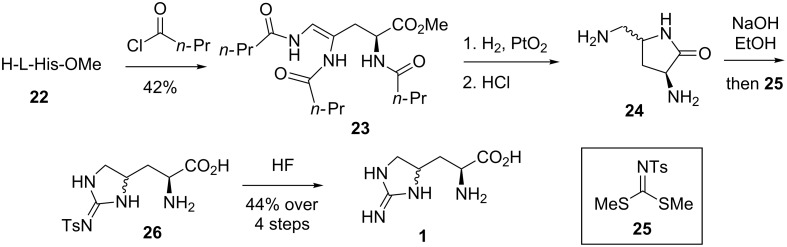
Synthesis of enduracididine (**1**) by Shiba et al.

**Synthesis of enduracididine by Shiba et al.:** The first diastereoselective synthesis of enduracididine (**1**) was reported by Shiba et al. in 1975 (Scheme 2) [54]. The synthesis began with Bamberger cleavage of L-methylhistidine (**22**) to afford amide **23**. Reduction of the double bond and cleavage of the three *n*-butyryl groups afforded lactam **24**. Lactam **24** was opened with base and directly treated with guanylating agent **25** giving tosylguanidine **26** which was unstable upon standing. Immediate treatment with anhydrous HF gave L-enduracididine (**1**) as a mixture of diastereomers.

**Scheme 3:**
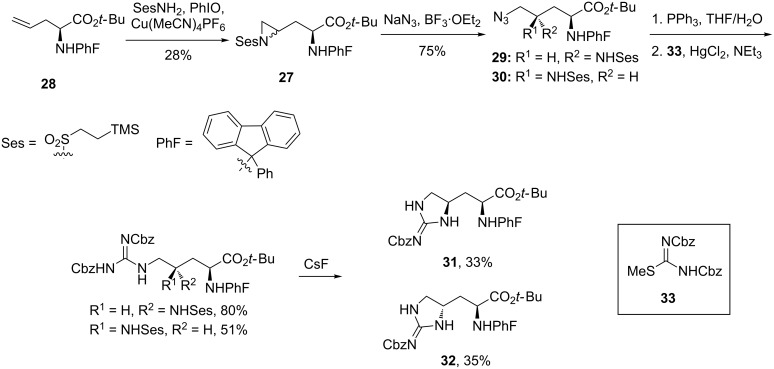
Synthesis of protected enduracididine diastereomers **31** and **32**.

**Synthesis of enduracididine and allo-enduracididine by Dodd et al.:** No further synthetic investigations were reported until 2004 when Dodd et al. published a synthesis of protected enduracididine using an azide ring opening of a chiral aziridine as the key step (Scheme 3) [55]. The 9-phenylfluorenyl (PhF) protecting group was employed to help prevent undesired copper coordination during the key aziridation step.

The synthesis relied on the stereoselective formation of aziridine **27**. This key reaction proceeded from allylglycine **28** in 28% yield to give a 7:3 mixture in favour of the *S*,*S* diastereomer. Attempted optimisation of the yield and diastereoselectivity afforded no improvement. The synthesis continued with aziridine opening using sodium azide and BF_3_·OEt_2_ in DMF at 65 °C, conditions which were key to prevent undesired intramolecular ring opening. The two diastereomers **29** and **30** could then be separated and elaborated to afford enantiopure protected L-enduracididine **31** and L-allo-enduracididine **32**.

**Scheme 4:**
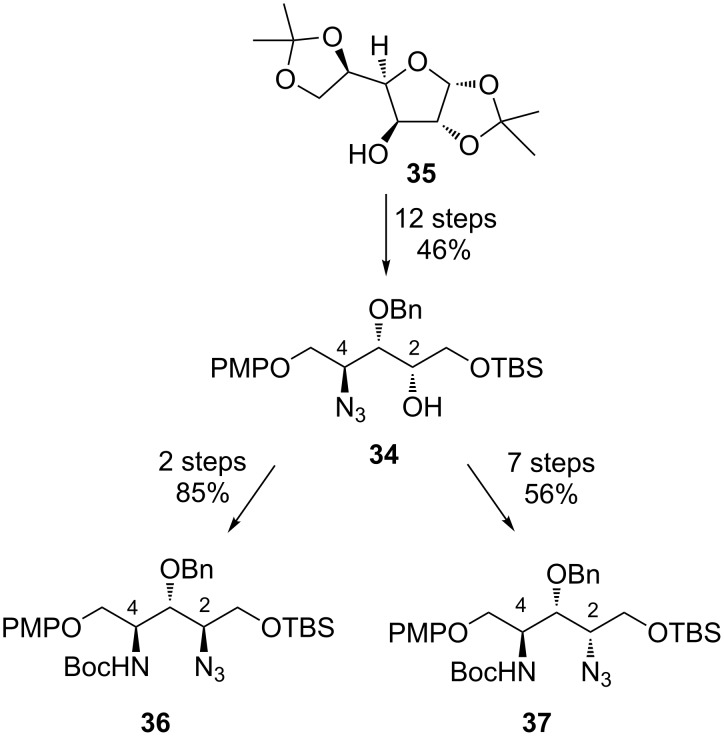
Synthesis of the C-2 azido diastereomers **36** and **37**.

**Synthetic studies towards β-hydroxyenduracididine by Oberthür et al.:** In 2009, Oberthür et al. reported a synthetic route to azide derivatives of β-hydroxyenduracididine [56]. The synthesis hinged on the use of azide **34** as a common intermediate to access both diastereomers. Diacetone D-glucose **35** was converted to azide **34** in 46% yield over twelve steps (Scheme 4). Azide **34** was easily converted to amino azide **36** via a two-step sequence, but conversion of azide **34** to amino azide **37** was more complex and required additional transformations [56].

**Scheme 5:**
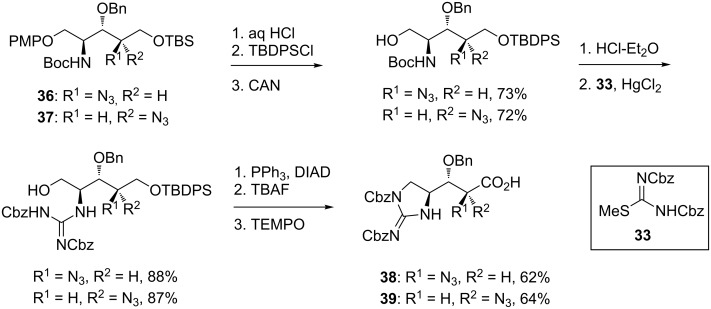
Synthesis of 2-azido-β-hydroxyenduracididine derivatives **38** and **39**.

Conversion of both amino azides **36** and **37** to azido acids **38** and **39** began with protecting group manipulation and installation of the guanidine using *S*-methylisothiourea **33** (Scheme 5). Mitsunobu cyclisation followed by deprotection and oxidation afforded the azido acids **38** and **39** in 40% yield over 8 steps from amino azides **36** and **37**.

**Scheme 6:**
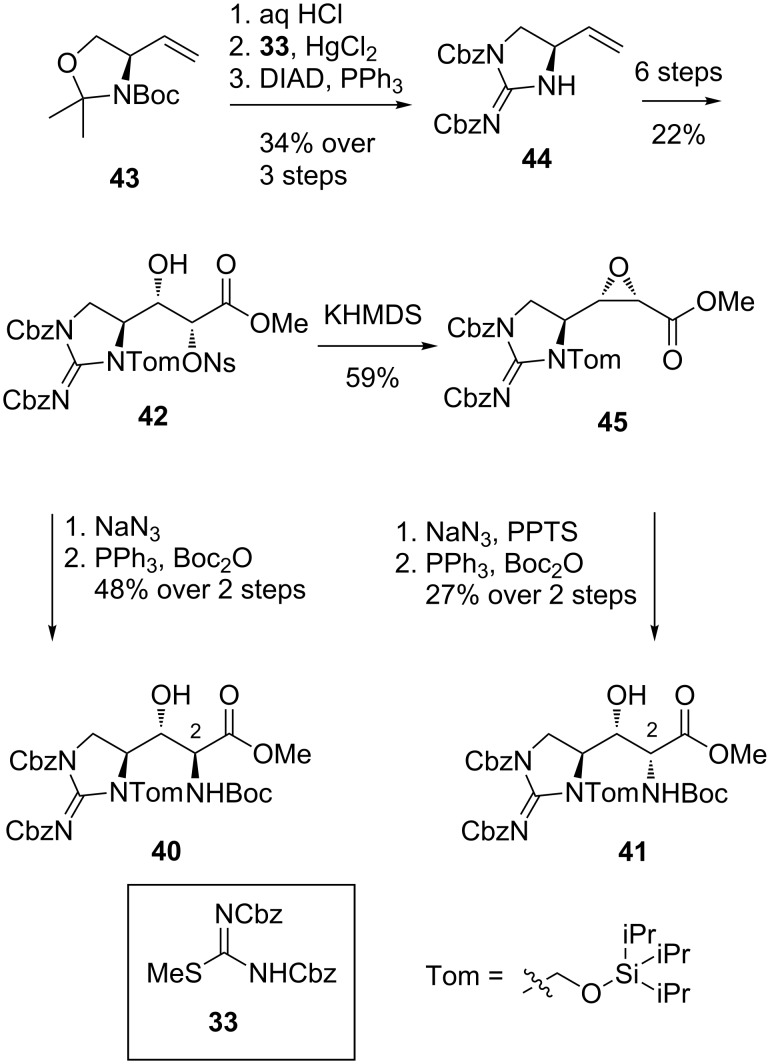
Synthesis of protected β-hydroxyenduracididine derivatives **40** and **41**.

**Synthesis of β-hydroxyenduracididine by Nieuwenhze et al.:** In 2010, Nieuwenhze and Oliver reported a synthesis of protected β-hydroxyenduracididines **40** and **41** making use of intermediate nosylamine **42** (Scheme 6) [57]. The synthesis of **42** began with alkene **43**, available from (*S*)-Garner’s aldehyde. Cleavage of the protecting group allowed installation of the guanidine group using isothiourea **33** before cyclisation was effected using Mitsunobu conditions. After a six-step conversion of alkene **44** to nosyl intermediate **42**, the synthesis diverged to access both C-2 diastereomers. Displacement of nosylate **42** with sodium azide followed by reduction and amine protection, afforded protected β-hydroxyenduracididine **40**. Alternatively, formation of epoxide **45** provided access to diastereomer **41**.

**Scheme 7:**
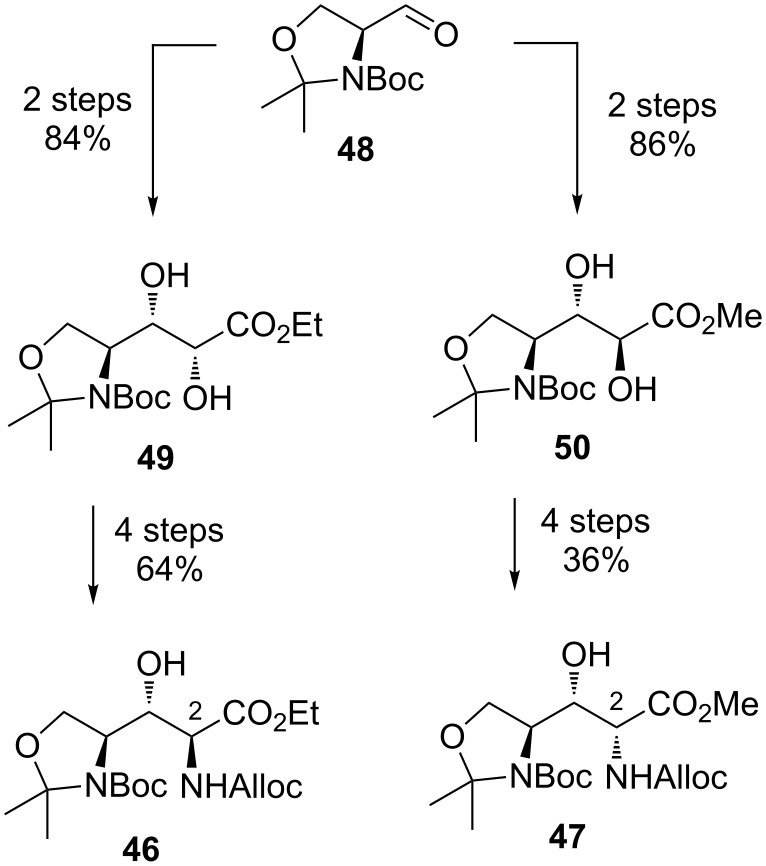
Synthesis of C-2 diastereomeric amino acids **46** and **47**.

**Synthesis of β-hydroxyenduracididine by Oberthür et al.:** In 2014, Oberthür et al. reported a second generation synthesis of β-hydroxyenduracididine using a more concise route to orthogonally protected amino acids **46** and **47** (Scheme 7) [58]. Installation of the C-2 stereocentre again began with Garner’s aldehyde **48** and Wittig olefination, followed by Sharpless dihydroxylation to stereoselectively afford diol **49** [59–60]. The C-2 epimer was accessed via Still–Gennari olefination of aldehyde **48** to afford the Z-olefin, which underwent dihydroxylation using potassium osmate to afford diol **50** [61–62]. With both diastereomers in hand, conversion to protected amino acids **46** and **47** was effected in four steps.

**Scheme 8:**
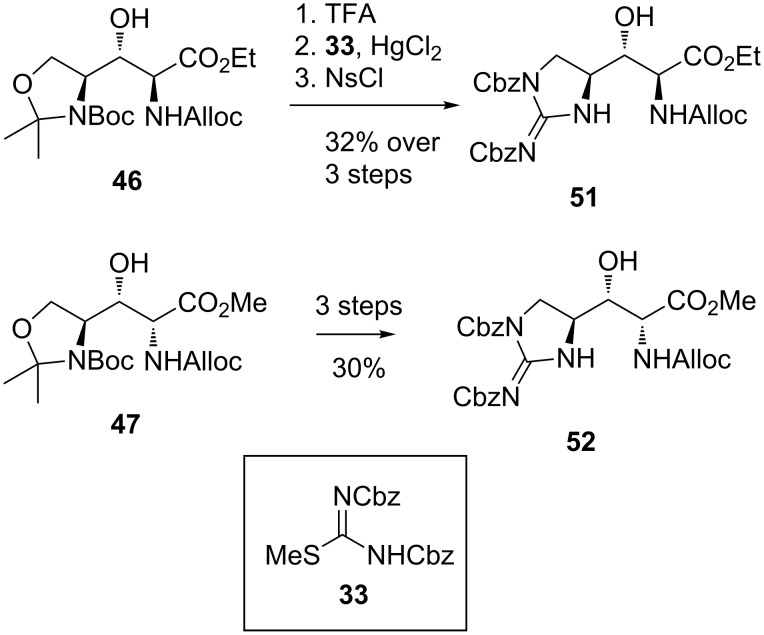
Synthesis of protected β-hydroxyenduracididines **51** and **52**.

With amino acids **46** and **47** in hand, conversion to the corresponding cyclic guanidines **51** and **52** was initiated through cleavage of the *N*,*O*-acetonide and guanylation using isothiourea **33** activated with HgCl_2_ (Scheme 8). Cyclisation of the guanidine afforded protected β-hydroxyenduracididine **51** in 21% yield in seven steps from diol **49**. The C-2 epimer **47** was converted to β-hydroxyenduracididine **52** using the same procedure. The new route proved more efficient than the previous report and provided access to both diastereomers suitably armed with orthogonal protecting groups.

**Scheme 9:**
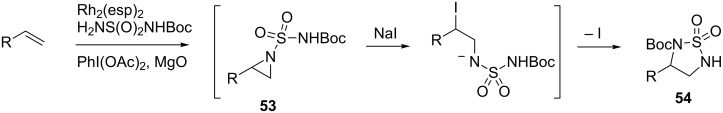
General transformation of alkenes to cyclic sulfonamide **54** via aziridine intermediate **53**.

**Synthesis of (±)-enduracididine and (±)-allo-enduracididine by Du Bois et al.:** The synthesis of (±)-enduracididine (**1**) and (±)-allo-enduracididine (**3**) reported by Du Bois et al. arose from the methodology for the conversion of alkenes to diamines via a cyclic sulfonamide intermediate using rhodium catalysis (Scheme 9) [63]. The reaction proceeds with formation of an intermediate aziridine **53** which rearranges upon addition of sodium iodide to afford the desired cyclic sulfonamide **54**.

**Scheme 10:**
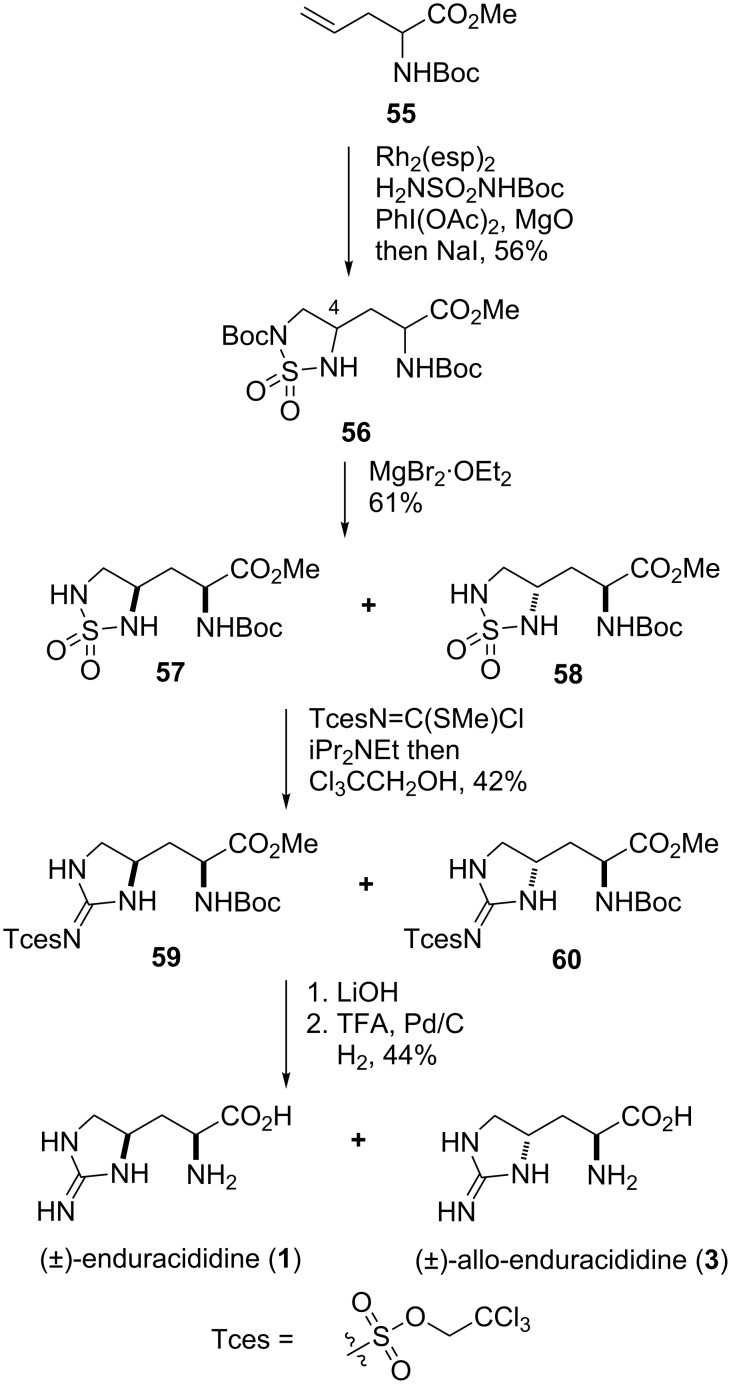
Synthesis of (±)-enduracididine (**1**) and (±)-allo-enduracididine (**3**).

For the synthesis of (±)-enduracididine (**1**) and (±)-allo-enduracididine (**3**), protected (±)-allylglycine **55** was treated with BocNHS(O)_2_NH_2_, MgO, Rh_2_(esp)_2_ and PhI(OAc)_2_ in isopropyl acetate followed by sodium iodide to afford cyclic sulfonamide **56** in 56% yield as a 1:1 mixture of diastereomers (Scheme 10). Selective deprotection of the sulfonamide Boc group allowed separation of diastereomers **57** and **58** via chromatography which were then converted to Tces (2,2,2-trichloroethoxysulfonyl) protected guanidines **59** and **60**. Global deprotection then afforded both (±)-enduracididine (**1**) and (±)-allo-enduracididine (**3**) in five steps and 6% yield from allylglycine **55**.

**Scheme 11:**
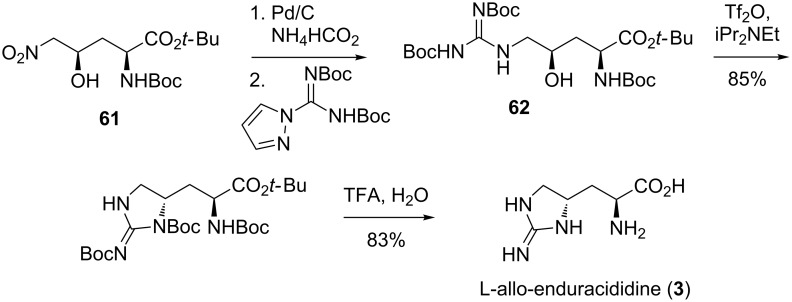
Synthesis of L-allo-enduracididine (**3**).

**Synthesis of L-allo-enduracididine by Ling et al.:** In 2014, Ling et al. filed a patent for their discovery of teixobactin (**17**) which included details of the structural elucidation. To confirm the configuration of the amino acids that are found in teixobactin, advanced Marfey’s analysis was performed, requiring samples of known absolute stereochemistry for comparison (Scheme 11) [64]. The synthesis of L-allo-enduracididine (**3**) was reported to begin with nitro alcohol **61** and afforded the free amino acid in four steps via key intermediate 4-hydroxyarginine **62**. The synthesis of nitro alcohol **61** was not described but its preparation has been reported [65]. All four diastereomers were synthesised for comparison with the isolated enduracididine sample.

**Scheme 12:**
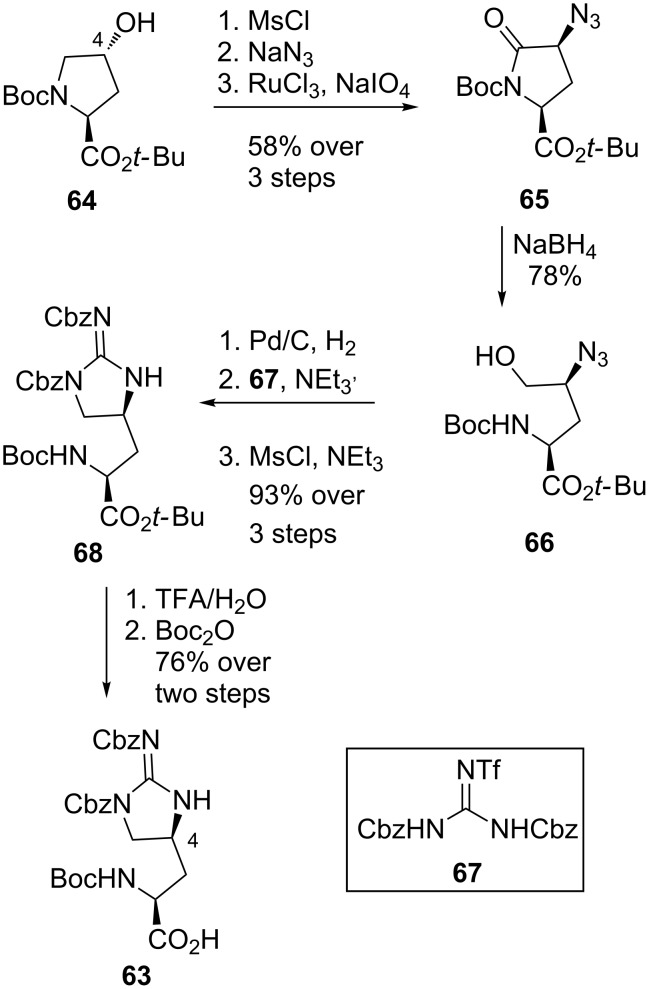
Synthesis of protected L-allo-enduracididine **63**.

**Synthesis of L-allo-enduracididine by Yuan et al.:** In 2015, Yuan et al. reported their synthesis of protected L-allo-enduracididine **63** from L-4-hydroxyproline **64** (Scheme 12) [66]. The C-4 stereocentre was installed through inversion of the hydroxy group of proline derivative **64** via mesylation and azide displacement to afford **65**. Oxidation installed the required carbonyl group which allowed reductive ring opening to afford alcohol **66**. Azide reduction, installation of the guanidine motif using Goodman’s reagent (**67**) [67] and cyclisation afforded **68**. Protecting group manipulation then afforded protected L-allo-enduracididine **63** with a free acid moiety available for peptide coupling over nine steps in 32% overall yield.

**Scheme 13:**
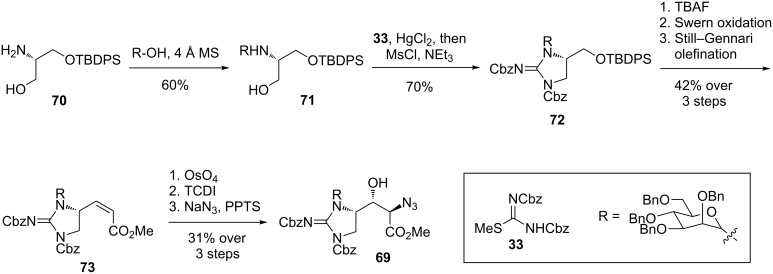
Synthesis of β-hydroxyenduracididine derivative **69**.

**Synthesis of a β-hydroxyenduracididine derivative by Cheng et al*.*:** In 2016, Cheng et al. reported work towards the *N*-mannosyl-D-β-hydroxyenduracididine (**69**) residue of the mannopeptimycins (Scheme 13) [68]. Their synthetic strategy started from silylated serinol **70** to which the mannosyl unit was attached to afford glycosylamine **71**, prior to construction of the cyclic guanidine motif and amino acid functionality. With glycosylamine **71** in hand, attention turned to installation of the guanidine moiety. Treatment of **71** with isothiourea **33** followed by mesyl chloride afforded cyclic guanidine **72** in 70% yield. Silyl deprotection, Swern oxidation and Still–Gennari olefination afforded *Z*-alkene **73**. Diastereoselective dihydroxylation of **73** followed by treatment with 1,1'-thiocarbonyldiimidazole (TCDI) and sodium azide afforded azide **69** over eight steps in 5.5% from silylated serinol **70**. The reported route was the most efficient of the many investigated however, the exact sequence of functional group installation was important in order to obtain high yields.

#### Synthesis of enduracididine-containing antibiotics

**Scheme 14:**
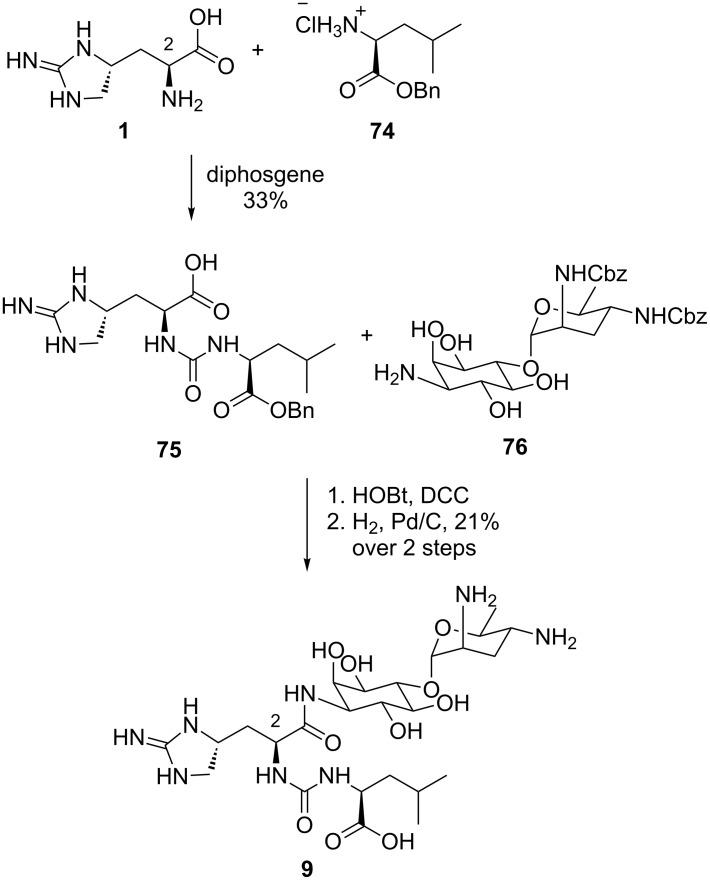
Synthesis of minosaminomycin (**9**).

**Synthesis of Minosaminomycin by Kondo et al.:** The only total synthesis of minosaminomycin (**9**) to date was reported in 1977 by Kondo et al. (Scheme 14) [69]. Enduracididine (**1**) was prepared using the method reported by Shiba et al. [54] and was coupled with the isocyanate formed in situ from protected leucine **74** affording urea **75**. Coupling of **75** with amino sugar **76** and global deprotection afforded minosaminomycin (**9**) in three steps from enduracididine (**1**). It should be noted that the diastereomer (2*R*-isomer) of **9** was also synthesised starting from D-enduracididine. Biological testing of both compounds revealed that the 2*R*-isomer exhibited 80% lower bacteriostatic activity against *Mycobacterium smegmatis* ATCC 607 compared to the parent natural product.

**Scheme 15:**
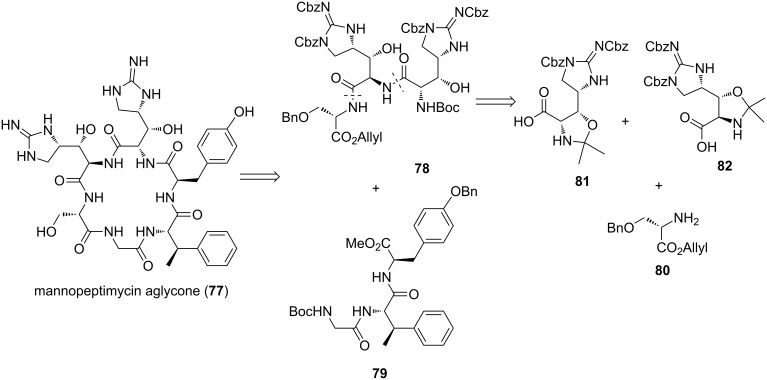
Retrosynthetic analysis of mannopeptimycin aglycone (**77**).

**Synthesis of Mannopeptimycin aglycone by Doi et al.:** In 2014, the total synthesis of the mannopeptimycin aglycone (**77**) was reported by Doi et al. [33]. The aglycone was synthetically broken down into tripeptides **78** and **79** (Scheme 15). Tripeptide **78** was further disconnected into protected serine **80** and protected β-hydroxyenduracididine residues **81** and **82**.

**Scheme 16:**
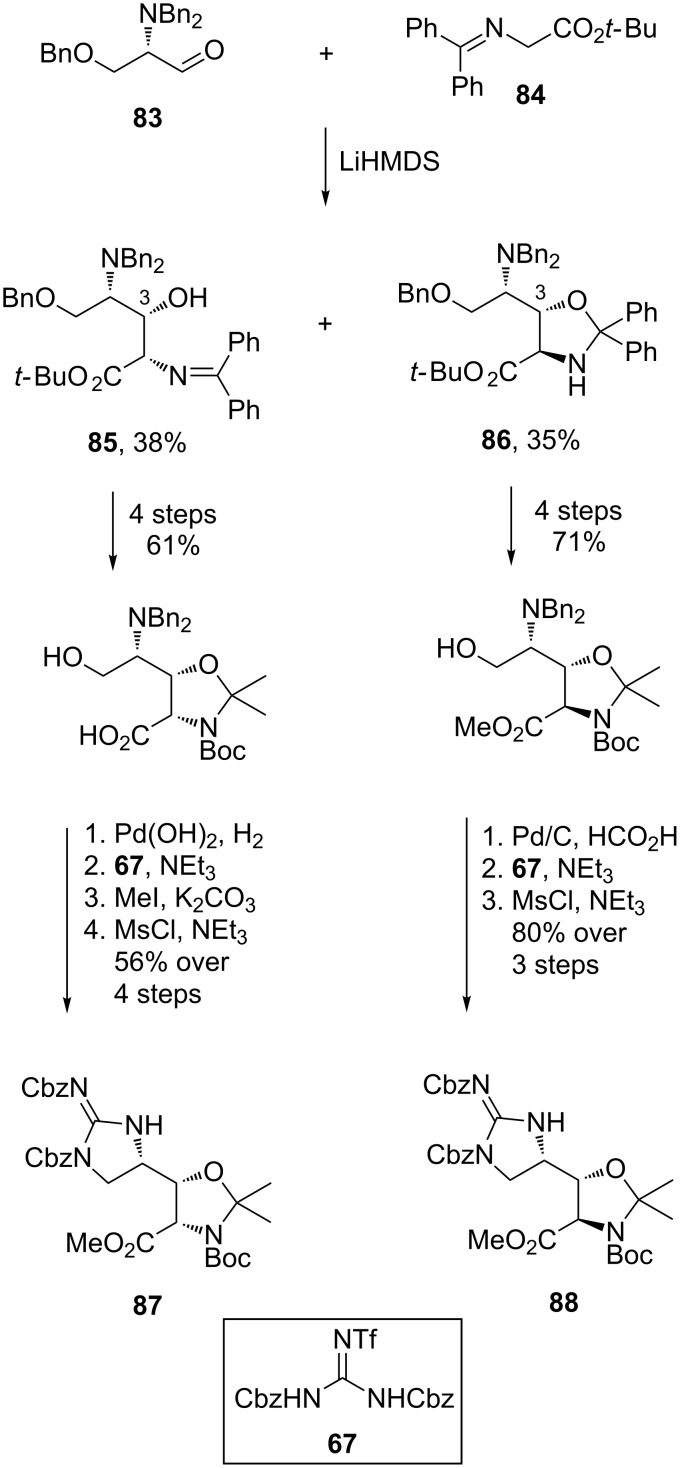
Synthesis of protected amino acids **87** and **88**.

The synthesis of key amino acids **81** and **82** was based on an aldol reaction between protected aldehyde **83** and glycine **84** (Scheme 16). The reaction yielded **85** and **86** which proved to be easily separated by chromatography. The C-3 stereochemistry of the addition products **85** and **86** was rationalised by the Felkin–Ahn model, and the inability of diastereomer **85** to cyclise due to unfavourable steric interactions. Conversion of aldol products **85** and **86** to acetals **87** and **88**, respectively, was achieved using standard transformations and Goodman’s reagent (**67**) [67] was used to install the guanidine moiety.

**Scheme 17:**
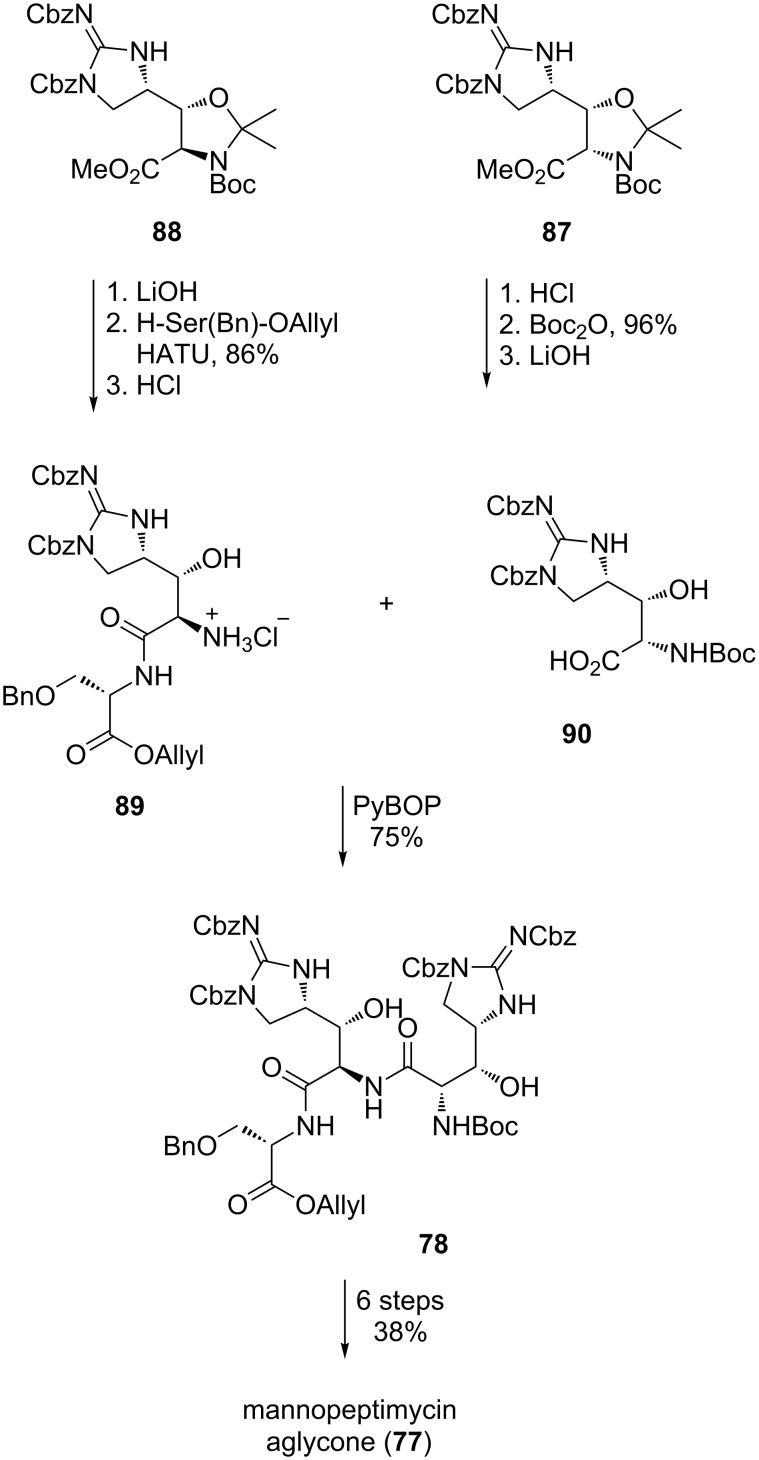
Synthesis of mannopeptimycin aglycone (**77**).

With both protected amino acids **87** and **88** in hand, the attention turned to the formation of tripeptide **78** (Scheme 17). Saponification of the ester of **88** and coupling with H-Ser(Bn)-*O*-Allyl and treatment with HCl afforded dipeptide **89**. A second peptide coupling with acid **90** then gave tripeptide **78**. With tripeptide **78** in hand, ligation with the remaining tripeptide **71** followed by cyclisation and global deprotection afforded the desired mannopeptimycin aglycone (**77**) in a further six steps and 38% yield from tripeptide **78**.

**Total synthesis of mannopeptimycins α and β by Chen et al.:** Chen et al. reported the first total synthesis of mannopeptimycins α (**12**) and β (**13**) in 2016 [70]. Previous biosynthetic and semisynthetic investigations had revealed that the *N*- and *O*-sugars of the natural products were essential for potent antibacterial activity [35,38–39,41]. The most difficult challenge involved the preparation of the *N*-α-mannosyl-D-β-hydroxyenduracididine unit. *N*-Mannosylation was complicated by steric hindrance around the reaction site and poor compatibility of the cyclic guanidine motif with Lewis acids.

**Scheme 18:**
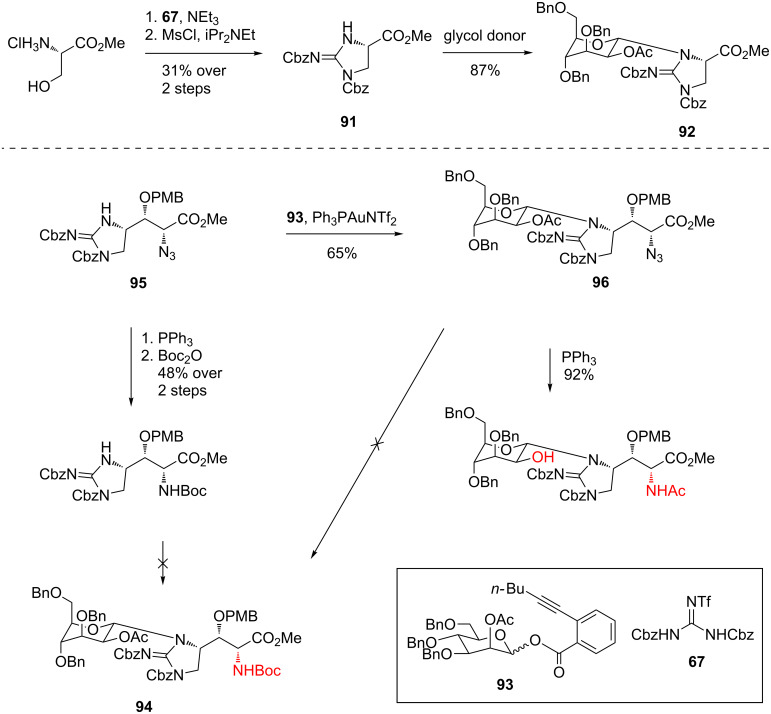
Synthesis of *N*-mannosylation model guanidine **92** and attempted synthesis of benzyl protected mannosyl D-β-hydroxyenduracididine **94**.

**Scheme 19:**
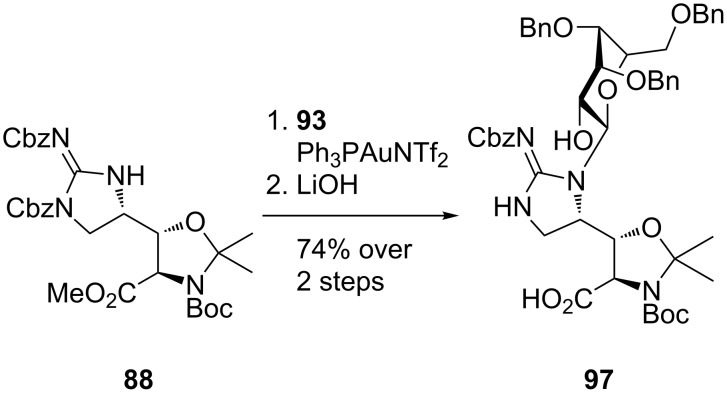
Synthesis of benzyl protected mannosyl D-β-hydroxyenduracididine **97**.

Initial attempts to glycosylate cyclic guanidine **91** using an array of donors under Lewis acidic or basic conditions failed to provide access to *N*-mannosylguanidine **92** (Scheme 18). However, gold(I) mediated [71] *N*-mannosylation using *ortho*-alkynyl benzoate **93** finally afforded *N*-mannosylated **92** in 87% yield [70]. Application of these conditions to afford the fully functionalised amino acid **94** was unsuccessful. However, encouraged by the successful *N*-mannosylation of azide **95** to afford adduct **96**, Chen et al. utilised the synthesis reported by Doi et al. [33] to prepare *N*,*O*-acetonide **88**. *N*-Mannosylation of acetonide **88** was successful and afforded the desired product in 86% yield (Scheme 19). Saponification then provided the desired benzyl protected mannosyl D-β-hydroxyenduracididine **97**.

**Scheme 20:**
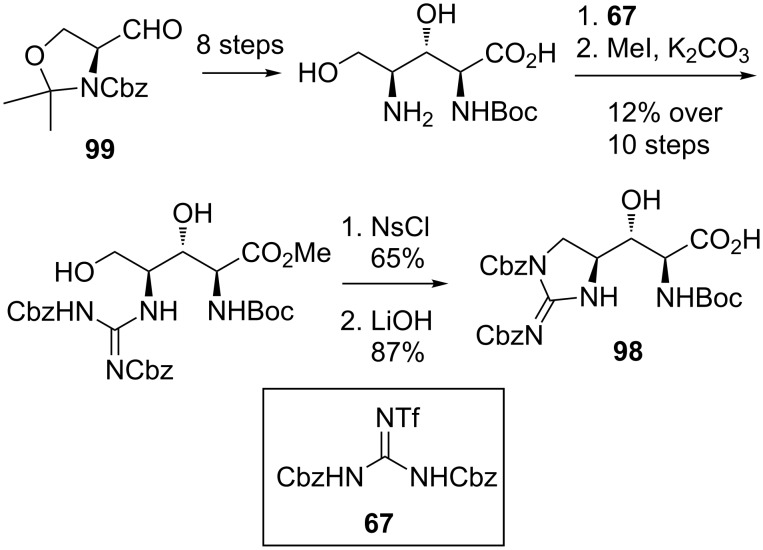
Synthesis of L-β-hydroxyenduracididine **98**.

Attempts to utilise the same procedure reported by Doi et al. [33] to provide the amino acid L-β-hydroxyenduracididine **98** were unsuccessful. An alternative route to L-β-hydroxyenduracididine based on the synthesis reported by Oberthür et al. [58] afforded L-β-hydroxyenduracididine **98** in 7% yield over twelve steps from **99** (Scheme 20).

**Scheme 21:**
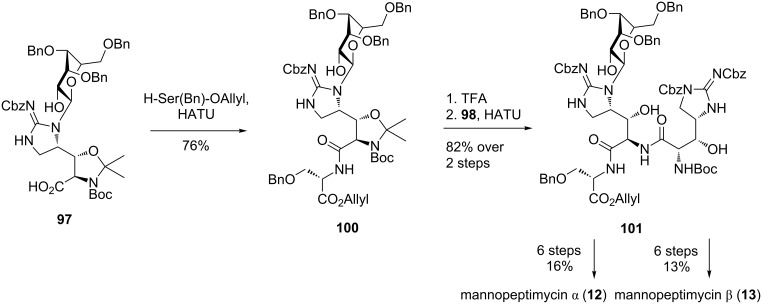
Total synthesis of mannopeptimycin α (**12**) and β (**13**).

In the final stages of the synthesis (Scheme 21), benzyl protected mannosyl D-β-hydroxyenduracididine **97** was coupled with H-Ser(Bn)-*O*Allyl to afford dipeptide **100**. Unmasking of the amino and alcohol functionalities and peptide coupling with L-β-hydroxyenduracididine **98** afforded tripeptide **101** with no loss of the sugar group. This then completed the synthesis of the key fragment **101** and both mannopeptimycin α (**12**) and β (**13**) could be accessed in a further six steps.

**Scheme 22:**
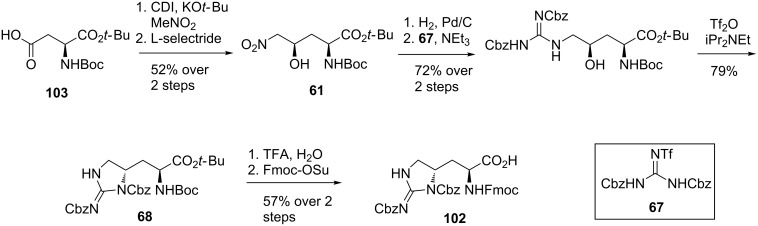
Synthesis of protected L-allo-enduracididine **102**.

**Synthesis of teixobactin by Payne et al.:** The first syntheses of the teixobactin framework were completed by Albericio et al. [46] and Singh et al. [47]. These syntheses substituted the enduracididine residue for the more readily available L-arginine. The total synthesis of the full teixobactin structure was completed in 2016 by Payne et al. [72] using Fmoc solid-phase peptide synthesis (SPPS). The key to the synthesis was access to the protected L-allo-enduracididine residue **102** (Scheme 22). The synthesis of this building block was achieved using a combination of reported procedures beginning with protected aspartic acid **103**. Using a protocol reported by Rudolph et al. [65] nitro alcohol **61** was accessed in two steps. Following procedures described in the patent filed by Ling et al. [64], alcohol **61** was converted to Boc-protected L-allo-enduracididine **68**. Protecting group exchange afforded the Fmoc protected L-allo-enduracididine **102** in seven steps and 17% yield from acid **103**.

**Scheme 23:**
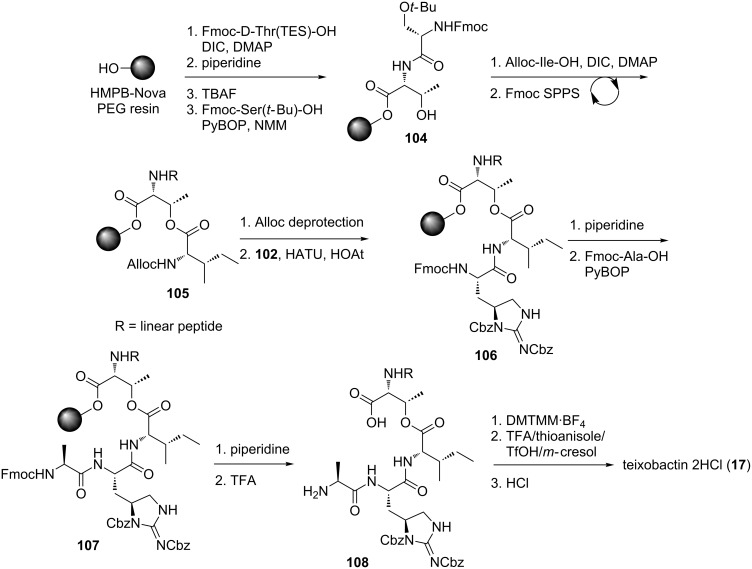
The solid phase synthesis of teixobactin (**17**).

The synthesis of the natural teixobactin (**17**) product began with Fmoc-D-Thr(TES)-OH on HMPB-NovaPEG resin. Successive couplings afforded peptide **104** (Scheme 23). Esterification with Alloc-Ile-OH and extension of the linear chain using conventional Fmoc SPPS afforded ester-peptide **105**. Deprotection of the *N*-alloc group and coupling of the key L-allo-enduracididine **102** residue proceeded smoothly giving resin bound peptide **106**. Brief (30 seconds) treatment of **106** with piperidine afforded the desired deprotected product, enabling coupling of the final amino acid. Extended exposure of peptide **106** to piperidine led to de-esterification. Final Fmoc removal of **107** and cleavage from the resin afforded linear peptide **108** which underwent macrolactamisation using 4-(4,6-dimethoxy-1,3,5-triazin-2-yl)-4-methylmorpholinium tetrafluoroborate (DMTMM·BF_4_) and acid-mediated global deprotection to afford teixobactin (**17**) in 3.3% yield over twenty four steps.

**Scheme 24:**
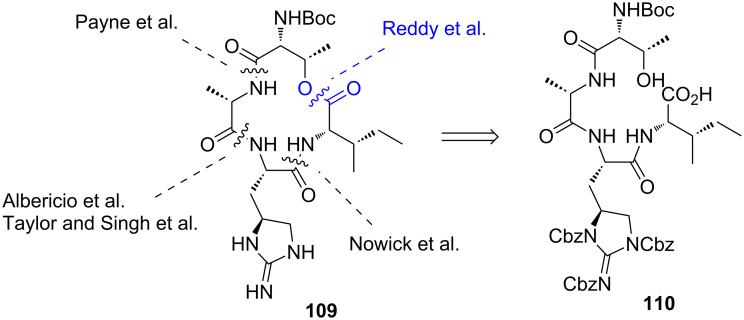
Retrosynthesis of the macrocyclic core **109** of teixobactin (**17**).

**Synthesis of the macrocyclic core of teixobactin by Reddy et al.:** In 2016, Reddy et al. reported their synthetic efforts towards teixobactin (**17**) with a solution-phase synthesis of the macrocyclic core **109** (Scheme 24) [73]. Their synthetic approach focused on the macrolactonisation of a linear precursor **110** differing from previous reports which employed macrolactamisation as the key ring-closing step [45–47,72].

**Scheme 25:**
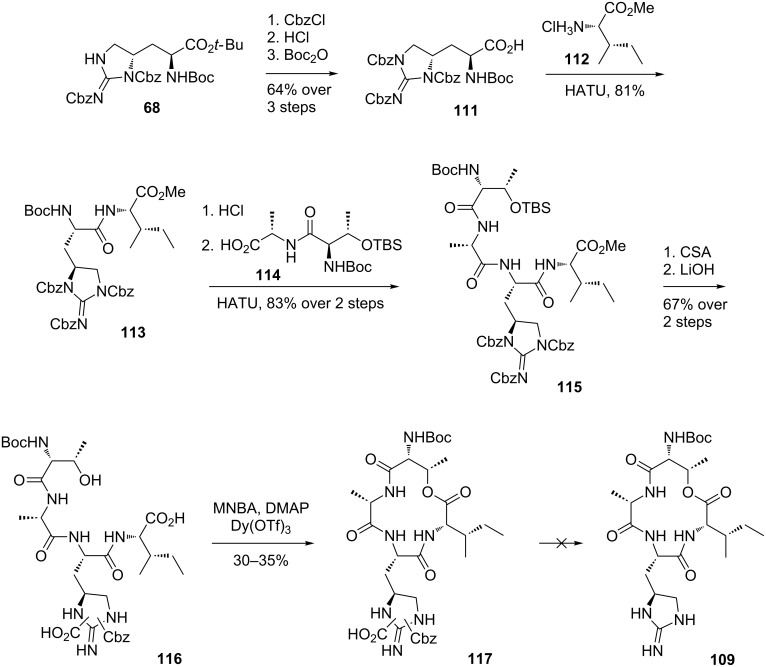
Synthesis of macrocycle **117**.

The synthesis of the linear precursor **110** began with protected L-allo-enduracididine **68**, which was prepared using procedures developed by Rudolph et al. and Peoples et al. (Scheme 25) [64–65]. The remaining NH of **68** was protected using Cbz-Cl before acid **111** was afforded after a two-step, deprotection-reprotection sequence. Fully protected enduracididine **111** was then coupled with L-isoleucine methyl ester **112** to give dipeptide **113**. Cleavage of the *N*-Boc group and coupling with dipeptide **114** afforded protected linear precursor **115**. Cleavage of the TBS and methyl ester protecting groups afforded seco-acid **116**. However, during the hydrolysis step, two of the three Cbz groups were cleaved from the enduracididine residue, and the position of the remaining Cbz and CO_2_H could not be determined. It was decided that final deprotection of the remaining enduracididine protecting groups would take place after formation of the macrocycle. Treatment of linear precursor **116** with modified Shiina macrolactonisation conditions reported by Batey et al. [74] of 2-methyl-6-nitrobenzoic anhydride (MNBA), DMAP and Dy(OTf)_3_ afforded macrocycle **117** in 30–35% yield. Unfortunately efforts to remove both the Cbz and CO_2_H moieties of **117** to afford macrocycle **109** under hydrogenation conditions were unsuccessful.

## Conclusion

The recent interest in teixobactin has resulted from its clinically unexploited mode of action, potent activity against resistant strains of bacteria and favourable pharmacokinetics. Structure–activity relationship studies of teixobactin suggest that the rare non-proteinogenic amino acid enduracididine, is a key residue for potent antibacterial activity. This observation has driven the need for new synthetic routes to enduracididine. However, current syntheses are cumbersome and inefficient. A robust and scalable synthetic route to an orthogonally protected enduracididine derivative suitable for solid phase peptide synthesis would greatly facilitate antibiotic drug development focused on a teixobactin inspired lead structure. Efficient access to enduracididine will enable ongoing structure–activity relationship studies of teixobactin and other lead compounds, for the development of much needed antibiotic drug candidates.
